# The MAHA clue - A case report

**DOI:** 10.1186/1757-1626-2-9385

**Published:** 2009-12-22

**Authors:** Vanamala Alwar, Karuna Rameshkumar

**Affiliations:** 1 Department of Clinical Pathology, St. John's Medical College Hospital, Bangalore -34, India

## Abstract

Microangiopathic hemolytic anemia (MAHA), is one of the causes of extra vascular hemolysis. It is seen in settings with pathologically altered small blood vessels. Disseminated carcinomas may rarely present as MAHA. A case of a 28 year old female with carcinoma stomach, who presented with MAHA as a first manifestation is reported. Acute onset of MAHA, may be the first manifestation of malignancy. In the absence of relatively common causes like disseminated intravascular coagulation,/Hemolytic uremic syndrome/thrombotic thrombocytopenic purpura, MAHA warrants extensive rapid investigations including bone marrow aspiration for possible metastatic deposits.

## Introduction

Brain, Dacie and Hourihane popularized the term Microangiopathic hemolytic anemia to designate anemias referring to red cell fragmentation occurring in association with small vessel disease [1]. MAHA is commonly encountered in clinical settings like- Hemolytic uremic syndrome (HUS), Thrombotic thrombocytopenic purpura (TTP) and Disseminated intravascular Coagulation (DIC). Rarely MAHA presents as a lethal manifestation of malignant tumours especially in mucin producing adenocarcinomas, but the pathogenesis of tumour associated mechanical hemolysis remains unclear. Many probable explanations have been put forth to explain the pathogenesis of this rare phenomenon:

(1) Some authors have proposed that *mucin *itself is directly responsible for the intravascular coagulation, and that the deposition of fibrin within microvasculature is in turn responsible for the red cell fragmentation.

(2) Another explanation is that the presence of tumour *microemboli *in pulmonary vasculature may lead to secondary intimal proliferation which in turn may be responsible for red cell fragmentation.

Unfortunately no effective or standard therapy is known for cancer associated MAHA. A small number of patients show a transient response to heparin. Hormonal or chemotherapy can also result in temporary remission [2].

## Case Report

A 28 year old female came with history of backache (Lumbar Region) for three weeks, fever and abdominal pain for 15 days and spontaneous ecchymotic patches over the lower limbs. She also gave history of loss of weight of approximately 10 kilograms over a period of one month. There was no history joint bleeds, bleeding per vagina or similar family or past history.

## On Examination

She had marked pallor and was febrile. Abdominal examination revealed, hepatomegaly (8 cm)and splenomegaly (8 cm). Sternal and spinal tenderness was noted over D7 and D10-11. The clinical diagnosis was a hematological malignancy in view of hepatosplenomegaly and rapid loss of weight.

## Laboratory Investigations

Hemoglobin 4.9 gm/dl:Total count: 17,000 cells/cmm

Differential count:Neutrophils:49%, Lymphocytes: 40%, Eosinophils: 3%, Monocyte:1%

Metamyelocytes:5%, Myelocytes:2% nRBCS: 19/100 WBCs

Platelet Count: 31,000/cmm

**Figure 1:**
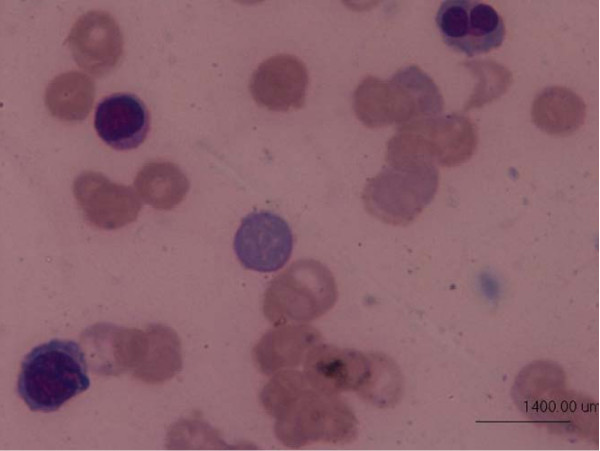
**Peripheral smear with presence of schistocytes, polychromatophilic cells and nucleated RBCs**. Leishman's stain × 1000.

The peripheral smear showed burr cells, schistocytes, spherocytes and nucleated RBCs which were consistent with features of MAHA (Figure 1).

**Figure 2:**
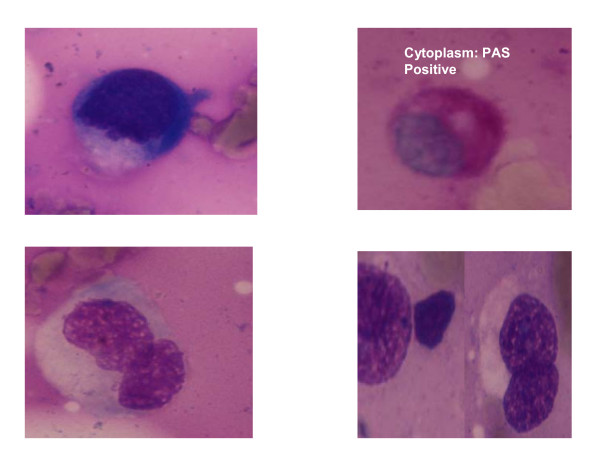
**The imprint smears showed presence of few large cells with foamy cytoplasm (Leishman's stainX1000) which stained PAS positive (PAS ×1000)**.

Bone Marrow done yielded a Dry Tap, however the imprint smears showed presence of few large cells with foamy cytoplasm.(Figure 2)

**Figure 3:**
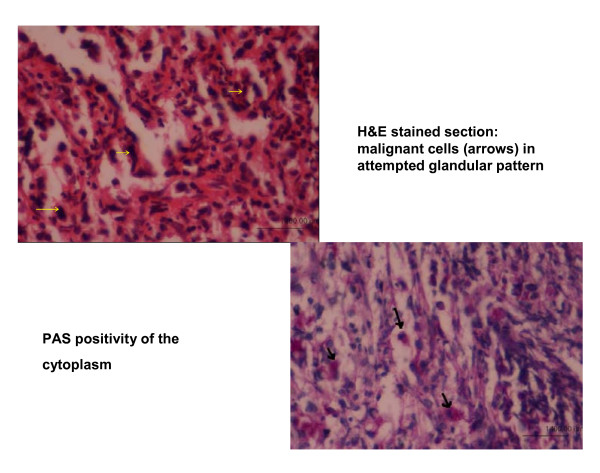
**Trephine biopsy done showed cells arranged in an attempted glandular pattern, suggestive of metastatic adenocarcinoma (Hemotoxylin and Eosin stain × 400)**. PAS stain showed positivity of the cytoplasm indicating the presence of mucin (Periodic acid Schiff × 400).

Trephine biopsy done showed cells arranged in an attempted glandular pattern, suggestive of metastatic adenocarcinoma. PAS stain showed positivity of the cytoplasm indicating the presence of mucin (Figure 3)

## Radiology

Lumbar X Ray, showed collapse of D12. Ultrasound examination of the abdomen showed para-aortic lymphadenopathy.

## Course in the hospital

During her hospital stay, she developed hematemesis (300 ml). Initial endoscopy was inconclusive due to improper visualization. Antral erosion was suggested as a probable cause. A repeat endoscopy done showed a stomal ulcer (1 × 1.5 cm) along the greater curvature. Biopsy was taken from the ulcer to rule out malignancy.

The histopathological evaluation of the biopsy from ulcer was reported as adenocarcinoma. The patient was given packed red cell transfusion for the anemia. Before a comprehensive treatment was established the patient opted for discharge against medical advice due to economical constraints.

## Discussion

MAHA is a rare complication of carcinomas and it can present as a paraneoplastic syndrome. Such patients may present with Coombs negative acute hemolytic anemia. MAHA may exist in two forms when associated with carcinoma:

1. May be associated with disseminated disease or

2. May be as a complication of chemotherapy, esp. with Mitomycin.

Both forms give rise to a severe hemolytic anemia and the latter is also associated with renal insufficiency [3]. Adenocarcinomas of the stomach, prostate, lungs in the order of occurrence are more commonly associated with MAHA compared to others. In a majority of cases the primary is not found [4]. Though a rare manifestation, the onset of MAHA heralds a lethal course within a few weeks [2]. It therefore warrants an immediate intervention. All efforts must be taken to find the primary tumour. Quick introduction of antineoplastic therapy, not only reduces the tumour bulk, but also causes remission of the MAHA. In cases where the primary was not found as reported by Lin et al, the anemia and thrombocytopenia responded dramatically to chemotherapy (5-furouracil, Mitomycin, Cisplatin) [5]. Plasmapheresis may also be useful.

## Conclusion

• Acute onset MAHA, may be the first manifestation of malignancy. In the absence of common causes (like DIC/HUS/TTP), it warrants extensive rapid investigations including bone marrow aspiration for possible metastatic deposits.

• Cancer associated MAHA is a life threatening condition with poor prognosis, warranting urgency in diagnosis and subsequent therapy.

## Consent

Written informed consent could not be obtained. The patient is anonymised and we believe the patient or their family would not object to publication of this case report.

## Competing interests

The authors declare that they have no competing interests.

## Authors' contributions

During the process of routine reporting, authors reported this case. VA wrote the article, KR edited the article and added the photographs to illustrate the contents.
